# Supplementary material for: An externally validated resting-state brain connectivity signature of pain-related learning
Source: Commun Biol. 2024 Jul 17;7:875. doi: 10.1038/s42003-024-06574-y (PMC11255216; doi:10.1038/s42003-024-06574-y)
Supplement: Supplementary file 2 — Supplementary Information [file 42003_2024_6574_MOESM2_ESM.pdf]

Supplementary Information: An externally validated resting-state brain connectivity signature of pain-related learning

Balint Kincses[1,2*], Katarina Forkmann[1], Frederik Schlitt[1], Robert Jan Pawlik[3], Katharina Schmidt[1], Dagmar Timmann[1], Sigrid Elsenbruch[2], Katja Wiech[4], Ulrike Bingel[1¶¶] & Tamas Spisak[1,2¶¶]

[1]Department of Neurology, Center for Translational Neuro- and Behavioral Sciences, University Medicine Essen, Essen, Germany
[2]Institute for Diagnostic and Interventional Radiology and Neuroradiology, University Medicine Essen, 45147 Essen, Germany
[3]Department of Medical Psychology and Medical Sociology, Faculty of Medicine, Ruhr University Bochum, Bochum, Germany
[4] Wellcome Centre for Integrative Neuroimaging, FMRIB, Nuffield Department of Clinical Neurosciences, University of Oxford, Oxford, UK.

[¶¶] These authors contributed equally

[*]**Corresponding author:**
Balint Kincses
Predictive Neuroimaging Laboratory & Bingel-laboratory

Department of Neurology

University Medicine Essen, University Duisburg-Essen

45147 Essen

Germany

e-mail: balint.kincses@uk-essen.de

Supplementary Methods

Paradigm

Participants performed a differential conditioning paradigm with two unconditioned stimuli. The experimental setting is visualized in *Supplementary Figure* 1. For more details, see the main text *Methods* part.

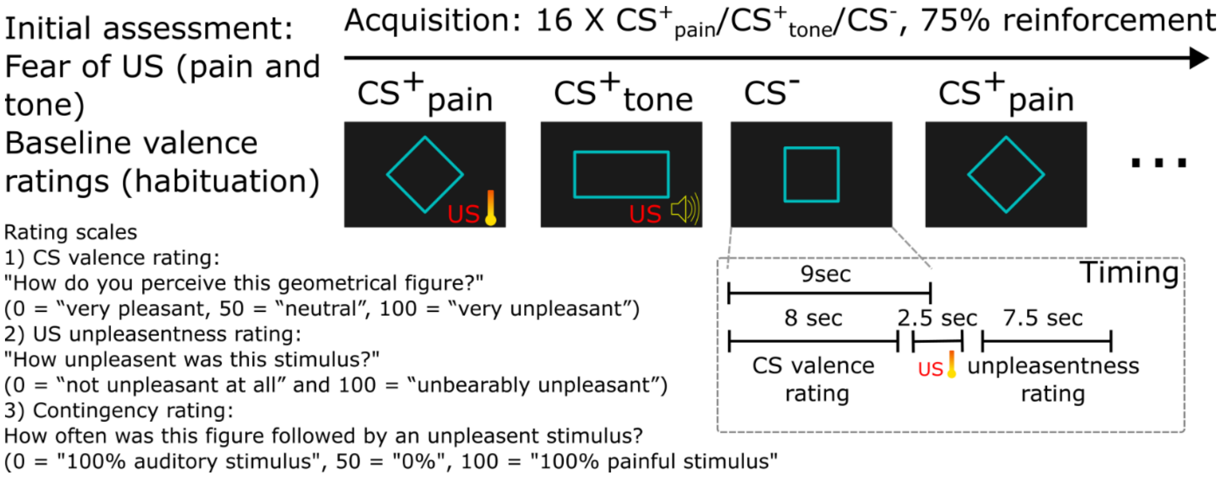

Supplementary Figure 1. The used paradigm in discovery sample and validation sample 1. In an initial assessment phase, participants rated their fear of the unconditioned stimuli (pain and tone) and the "baseline" valence of the geometric figures (habituation phase, not shown in the figure). An acquisition phase followed with 16 stimuli (each CS) with a 75% of reinforcement rate. An extinction phase followed the acquisition phase, in which the CSs were not associated with USs anymore (not shown). Different rating scales were used. CS valence ratings and US unpleasantness ratings were measured during acquisition and extinction after every 4th US pain or tone presentation, respectively. Contingency ratings were measured after acquisition and extinction. The figure was reproduce based on[1,2]. A similar paradigm was used in validation sample 2, but visceral pain was used instead of heat pain. The rating scale anchors differed (see Pawlik et al[3] for more detail.)

Calibration of aversive stimuli

In the first part of the calibration, individual thresholds for heat pain and the unpleasant tone were determined with the method of limits. For heat pain, the temperature was slowly increased (1°C/s) starting at a baseline of 35°C and the participants had to indicate when the sensation had become painful for the first time by pressing a button. The upper limit for heat stimuli was set to 50°C. A similar method was used to calibrate the auditory stimuli. Participant had to indicate when the gradually increasing loudness became unpleasantly loud. A maximum loudness of 120dB was never exceeded. Both procedures were repeated three times and the average value was used as heat pain and unpleasant tone threshold. The participants then received ten stimuli of different temperature levels two times (ranging from (pain threshold - 1°C) to (pain threshold + 3.5°C) with 0.5°C degree increments) resulting in 20 stimuli in total. After each stimulus, unpleasantness was rated. A linear regression model was fitted to the data to determine the temperature level corresponding to VAS70. This level was subsequently used in the matching procedure.

For visceral pain, different pressure levels (increments of 5 mmHg) with a duration of 30s were used to determine the visceral pain threshold. Participants provided the perception of the stimulus on a scale from 1 to 4 (1 = no perception over 2 = likely perception and 3 = urge to defecate to 4 = painful perception). The pressure was increased until the visceral pain threshold was reached (rating of 4). The upper pressure limit was set to 55 mmHg. The procedure was terminated when the participant reported the first sensation of pain. During the calibration, rectal distension stimuli delivered in 5 mmHg increments and rated until the predefined level (between VAS60-80) was reached. The starting pressure was 5 mmHg below the visceral pain threshold.

Supplementary Notes

Assessment of learning

We derived the preregistered differential valence change for pain as our main outcome measure (see *Equation 1* in the main text). Derivates of differential valence change for tone and differential valence change in the extinction for pain and tone were calculated as well. Additionally, to check the validity of our derived parameter, its relation to more complex measures of learning was estimated in the discovery sample. Namely, using a Rescorla-Wagner model, individual learning parameters were estimated and their correlation with our outcome variable was investigated (*Supplementary Figure 2*). We found considerable significant correlation between the learning rate parameter and the differential valence change (r=0.77, p<0.001 and r=0.92, p<0.001, when the fit was appropriate).

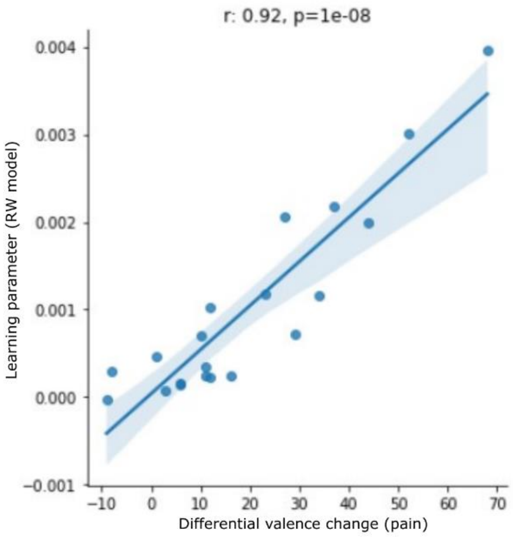

Supplementary Figure 2. Correlation between learning parameter (derived from Rescorla-Wagner model) and our predefined outcome variable in participants in which the model fit was appropriate.

The behavior results and the model performance in each study separately

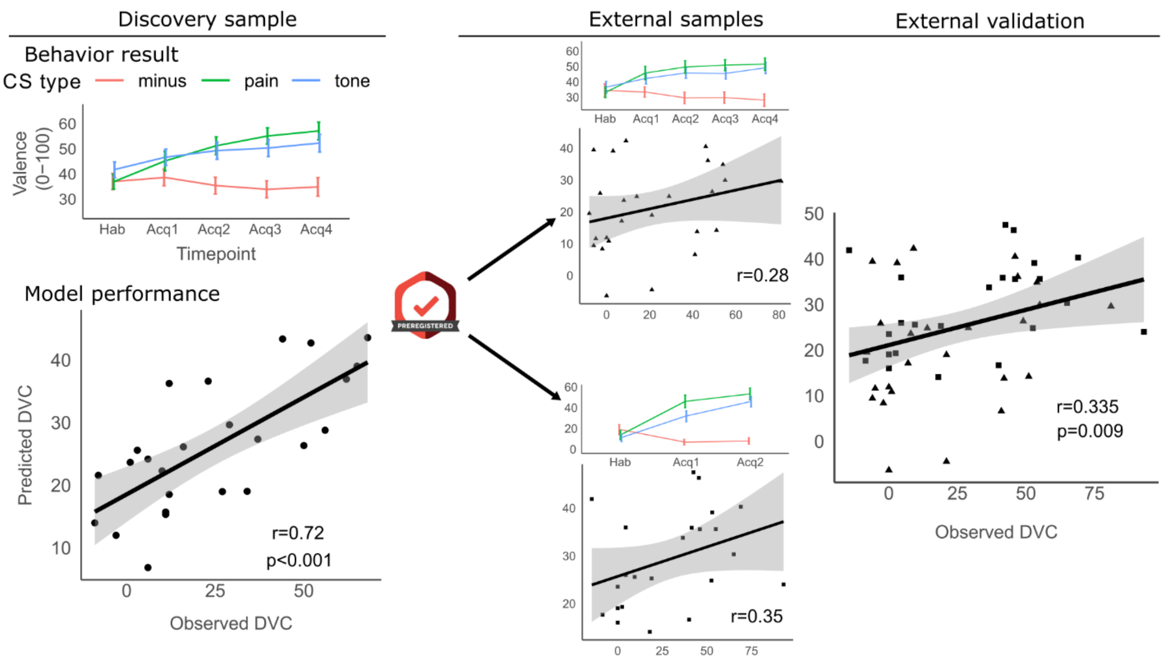

Supplementary Figure 3. Model development and validation.
In all three datasets, we observed an increase in valence ratings of the pain- and tone-related conditioned stimuli (CS^{+}_{pain} and CS^{+}_{tone}, respectively), as compared to the safety signal (CS^{-}), indicating successful conditioning. Mean and standard error of the ratings in each experimental phase is depicted by line plots, separately for all three datasets. Our model successfully predicted individual differential valence rating (DVC, see Equation 1) both in the discovery sample (r=0.72, p<0.001) and – after fixing and pre-registering all model parameters - in the external validation samples (r=0.34, p=0.009). Note that the behavior results are from all subjects before exclusion. The same pattern can be observed after exclusion (see main text *Figure 1b, Behavior result*).

Model performance on the extended sample

Including participants with high motion during the resting state scan (additional n=11, ~20% of subjects) resulted in similar effect size (r=0.35, p=0.003). That is, the correlation between predicted and observed differential valence change was significant, even if we included participants with the preregistered motion cut-off value (*Supplementary Figure 4*).

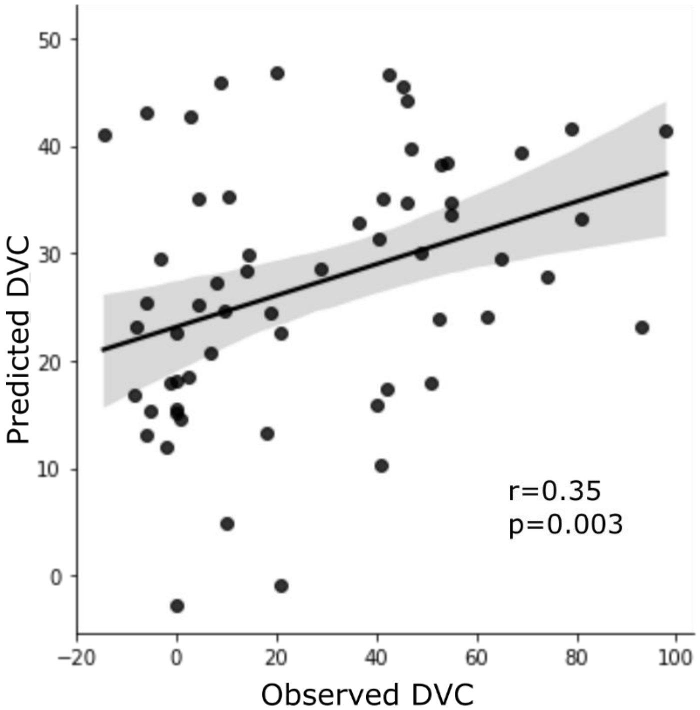

Supplementary Figure 4. Correlation between observed and predicted DVC values in the whole sample.
That is, participants with high motion during resting state scan are included as well. An additional 11 subjects (~20% of subjects) were included in the analysis. Including participants with high motion did not affect the effect size estimate.

Model training on tone related learning as target

For exploratory purposes, we also explored a model training to predict individual tone related learning. However, we observed that the discovery sample lacked sufficient power to achieve significant predictive performance for aversive tone learning using nested cross-validation (see the project github page). That is, the result of cross validation procedure was non-conclusive and the fitted model was not capable to predict tone related learning in the validation datasets (see scatter plots of validation dataset 1,2 in the github page). It is important to note that the lack of power does not affect the robustness of our findings about our model for pain-related learning, as those results were evaluated in a pre-registered external validation, following the best practices for small sample model discovery[4,5]. While our pre-registered external validation is well positioned to confirm our true positive discoveries (predictive model of pain-related learning), it is not able to confirm whether the lack of significant predictions for tone-related learning is a false negative observation[5].

Model validators in the validation sample

We repeated the specificity analysis on the validation samples (see main text: *Specificity to pain-related learning and confounding bias* for more details).

Extinction learning: The RCPL score was correlated with differential valence change in the extinction phase (R^2_{VS1}=3.8%, p=0.17; R^2_{VS2}=12.8%, p=0.045; $R^2_{VS1+VS2}$=11.2%, p=0.008). However, accounting for the baseline association between acquisition and extinction learning (R^2_{VS1}=42%, p=<0.001; R^2_{VS2}=71.6%, p<0.001; $R^2_{VS1+VS2}$=58%, p<0.001), we have found no evidence with the partial generalization test that this association was more than a consequence of this baseline correlation (p_{VS1}=0.54, p_{VS2}=0.62, $p_{VS1+VS2}$=0.33 for the validation sample 1, validation sample 2, and merged sample, respectively) (*Supplementary Table 6*). Therefore, we found no evidence that the association with extinction was more than a consequence of the correlation between acquisition and extinction learning in the validation samples.

Tone learning: The RCPL score was correlated with aversive tone related leaning, but only significantly in the merged sample (R^2_{VS1}=7.4%, p=0.09; R^2_{VS2}=2.6%, p=0.23; $R^2_{VS1+VS2}$=7.8%, p=0.025). However, again the partial generalization test indicated that this association was actually a secondary consequence (p_{VS1}=0.32, p_{VS2}=0.82, $p_{VS1+VS2}$=0.35) of the baseline association between the two types of learning (R^2_{VS1}=41%, p<0.001; R^2_{VS2}=63%, p<0.001; $R^2_{VS1+VS2}$=53%, p<0.001) (*Supplementary Table 6*).

Contingency learning: In the two validation samples, contingency awareness was measured slightly differently, namely, the anchors of the used scale were different (see *Methods* for detail). Therefore, we investigated the association between the model prediction and contingency awareness separately. In validation sample 1, RCPL score was associated with contingency rating for the CS^- (R^2_{VS1}=18.5%, p=0.014), but not with contingency ratings for the CS^+_{pain} (R^2=0.3%, p=0.4). This association of CS^- with model prediction was significant (R^2_{VS1}=18.5%, p_{VS1}=0.04) and higher than expected from the association between valence-learning and contingency learning (R^2_{VS1}=11.6%, p=0.04) and the actual predictive performance to valence learning (R^2=7.7%, p=0.084). Therefore, the model generalized to contingency awareness for CS^- after acquisition. In validation sample 2, contingency scales were used differently (see Pawlik et al.[3] and Koenen et al.[6] for more detail). Therefore, we only investigated the association with contingency awareness for CS^+_{pain}, however, it was not correlated with RCPL score (R^2=2.9%, p=0.22) (*Supplementary Table 6*).

Power calculation for specificity analysis

The power calculation is based on simulated data (see Spisak et al.[7] for details). A multivariate normal distribution was sampled 100 times to generate simulated target variable (y; i.e. learning performance), model predictions ($\hat{y}$) and validator or confounder variable (c; e.g. learning rate or tone-related learning performance) for n=49 simulated participants. We set the association between the target and predictions to $R^2_{y\hat{y}} = 11.6\%$, in line with the observed model performance. We assumed that the model explains a large, but realistic amount of variance in the validator (or confounder): $R^2_{c\hat{y}} = 40\%$. We were interested in the effect of the association between the target and the validator (confounder) on statistical power. Thus, we investigated the following values of R^2_{cy} : 10, 20, 30, 40, 50, 60, 70, 80 and 90%. For each of these values, in each of the 100 repetitions, we performed partial confounder testing with the package '*mlconfound*', with the number of permutations set to N_{perm}=1000. Our results show that 80% power is achievable only of $R^2_{cy} < 50\%$ (*Supplementary Figure 5*). Thus, in the present study, our test can be expected to provide sufficient power when testing specificity to pain against tone-related learning (divergent validator) ($R^2_{cy} = 49\%$) or generalization to extinction learning (convergent validator) ($R^2_{cy} = 46.1\%$), but not when testing for generalizability to learning rate ($R^2_{cy} = 84.6\%$). Blue and red lines on the figure denote statistical power as a function of target-validator association and 80% power, respectively.

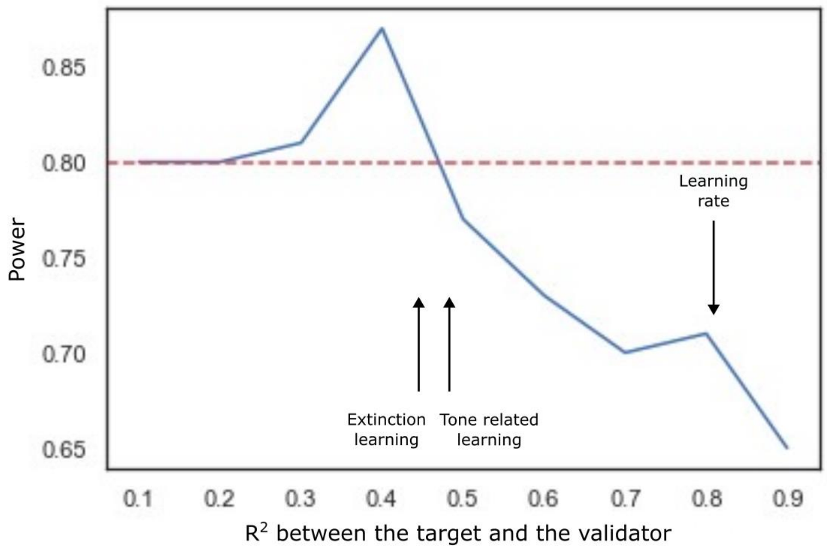

Supplementary Figure 5. Power calculation for specificity analysis.

The location of an example ROI (amygdala).

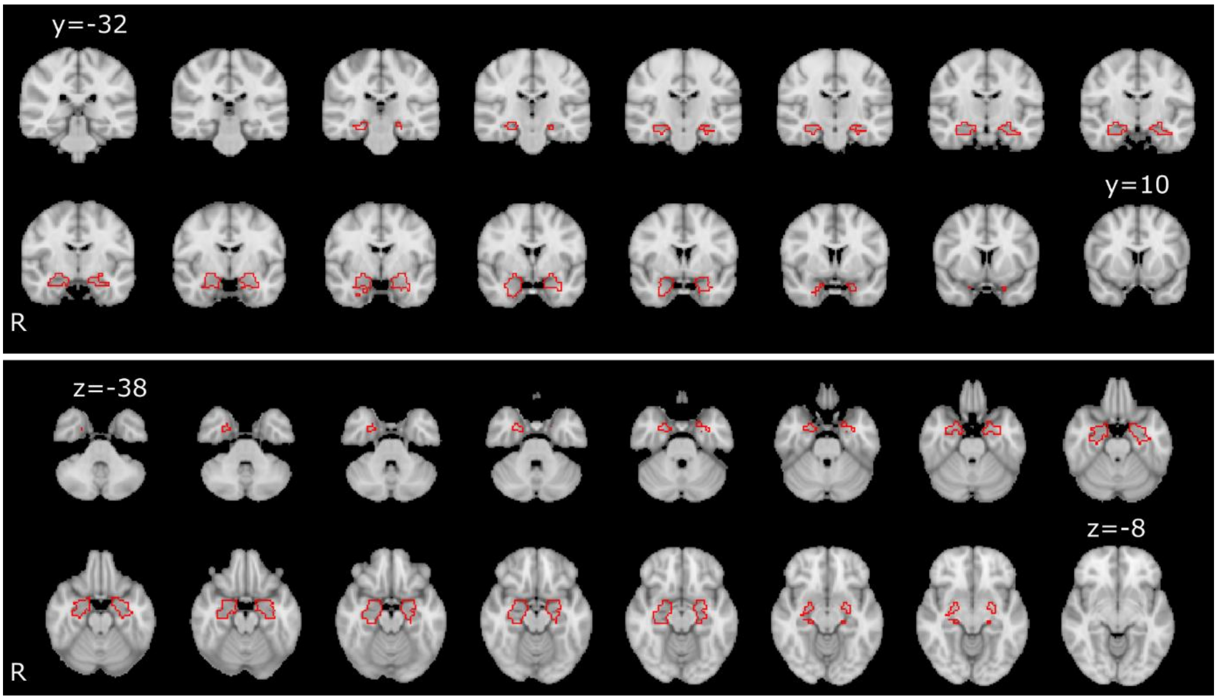

Supplementary Figure 6. The outline of the applied functional amygdala region in the coronal and axial slices of MNI space.

Note that the used atlas was based on functional parcellation, therefore the anatomical boundaries might be violated. The used regions were back-projected to the individual functional space and intersected with the gray matter mask to minimize inaccuracies introduced by registration. The number of the initial and last slices in each orientation is shown, intermittent slices are sampled evenly. The location of each region can be found in *Table 3* and *Supplementary Table 7*.

Sample name	Discovery sample	Validation sample 1	Validation sample 2
N(after exclusion/total)	25(38)	26(33)	23(28)
Age	24.2 (3.9)	25.7 (4.3)	25.4 (3.7)
Sex (female/male)	11/14	16/10	13/10
Pain stimulation	Heat pain	Heat pain	Visceral pain
Conditioning task	In-scanner	Out-of-scanner	In-scanner
Days between MRI and task	0	Median: 3(range: 1-5)	0
Other comment	-	-	10(11) participants received placebo injection after the resting state fMRI, but before the task
Headcoil # channels	20	32	32
Resolution (mm)	2.5x2.5x3		3x3x3
TR (ms)	2300		2500
TE (ms)	28		30
distance factor (%)	15		0
Total volumes	260		192

Supplementary Table 1. Factors contributing to the heterogeneity between samples.
All the samples were acquired in the same 3T scanner (Siemens Magnetom Skyra), but different personal. The main difference was the time between the resting scan and the behavior task, namely in the discovery sample and validation sample 2, the task was performed in-scanner, on the same day. While in the validation sample 1, the task was performed out-of-scanner on a different day.

		Variable name	Discovery sample	Validation sample 1	Validation sample 2
valence		$\Delta(\Delta CS^{+}_{pain}, \Delta CS^{-})_{ext}$	-21.4 (27.1)	-8.2 (16.6)	-20.6 (23.4)
		$\Delta(\Delta CS^{+}_{tone}, \Delta CS^{-})_{ext}$	-18.6 (26.1)	-9.8 (17.5)	-22.1 (19.6)
contingency		CS^{+}_{pain} acquisition	56 (40)	50 (49)	66 (38)
		CS^{+}_{tone} acquisition	21.9 (54)	25 (44)	71 (36)
		CS^{-} acquisition	-2 (21)	10 (19)	NA
		CS^{+}_{pain} extinction	14 (34)	3 (18)	6 (15)
		CS^{+}_{tone} extinction	19 (31)	1 (21)	6 (16)
		CS^{-} extinction	-5 (16)	2 (14)	NA
US unpleasantness	habituation	pain	59 (28)	69 (15)	-
		tone	50 (19)	54 (15)	-
	acquisition	pain	54 (16)	52 (16)	74 $(22)^{++}$
		tone	51 (17)	51 (16)	62 $(23)^{++}$

Supplementary Table 2 Mean (and standard deviation) values of corresponding measures of included subjects.

ΔCS^{+}_{pain} –valence change between end of extinction and end of acquisition for the conditioned stimulus associated with pain, ΔCS^{+}_{tone} –valence change between end of extinction and end of acquisition for the conditioned stimulus associated with aversive tone, ΔCS^{-} - valence change between end of extinction and end of acquisition for the conditioned stimulus. associated with no aversive stimulus. Contingency measured after acquisition and after extinction, reflecting the cognitive aspect of learning. Reported US unpleasantness in the habituation and acquisition. $^{++}$ -Unpleasantness measured after the first half and end of the acquisition in validation sample 2 as compared to the discovery sample and validation sample 1, where unpleasantness was also measured before acquisition and during acquisition.

Variable name	Discovery sample	Validation sample 1	Validation sample 2
Age	24.2 (3.9)	25.7 (4.3)	25.4 (3.7)
Mean FD	0.085 (0.029)	0.083 (0.021)	0.078 (0.022)
Median FD	0.073 (0.025)	0.073 (0.017)	0.067 (0.021)
Max FD	0.54 (0.44)	0.4 (0.23)	0.48 (0.47)
Percent of scrubbed volumes	9.5 (7.4)	8.5 (7)	7.74 (10)
Fear of pain	30.6 (23.7)	35.5 (26)	N.A.
Fear of tone	15.8 (11.5)	15.5 (18.2)	N.A.
Difference of US fear	14.8 (19.8)	20 (19)	N.A.
PASS_D1	2.4 (1)	2.1 (1.1)	N.A.
PASS_D2	1.8 (0.8)	1.6 (1.1)	N.A.
PASS_D3	1.3 (1)	0.96 (0.8)	N.A.
PASS_D4	1.2 (0.8)	1.2 (0.9)	N.A.
PCS	19 (8.9)	13.8 (10.3)	N.A.
ADS-K	7.9 (7.2)	9.2 (8.8)	N.A.

Supplementary Table 3. Confounders measured in all samples.
Additional demographical, psychometric, and in-scanner motion were measured. None of the investigated confounders bias the model. All values represent the mean and standard deviation of the sample used for the main analysis. FD – framewise displacement, PCS, Pain Catastrophizing Scale; PASS D1, Pain Anxiety Symptoms Scale, subscale Cognitive; PASS D2, subscale Escape/Avoidance; PASS D3, subscale Fear; PASS-D4 subscale Physiological Anxiety, ADS-K –Center for Epidemiological Studies-Depression scale

	PAIN		AVERSIVE TONE	
	Valence	Contingency*	Valence	Contingency*
	$\Delta(\Delta CS^{+}_{pain},\Delta CS^{-})$ (95% CI)	CS^{+}_{pain} (95%CI)	$\Delta(\Delta CS^{+}_{tone},\Delta CS^{-})$ (95%CI)	CS^{+}_{tone} (95%CI)
Discovery Study (n=38)	22 (12-31)	50 (35-65)	12 (4-22)	25 (9-41)
Validation Study (n=61)				
sub-sample 1 (n=33)	24 (15-35)	49 (33-63)	19 (9-28)	17 (1-33)
sub-sample 2 (n=28)	29 (20-40)	68 (55-88)*	27 (18-36)	72 (60-84)*

Supplementary Table 4. Behavioral results from all subjects (before exclusion).
Results show evidence for threat learning in both the pain and the unpleasant tone conditions, in all three studies. * Note that the contingency ratings were measured with different scales in the discovery and validation sample 1 as compared to validation sample 2. Values represent the mean and bootstrapped confidence intervals value in parenthesis. ΔCS^{+}_{pain} –valence change between end of acquisition and habituation phase for the conditioned stimulus associated with pain, ΔCS^{+}_{tone} –valence change between end of acquisition and habituation phase for the conditioned stimulus associated with aversive tone, ΔCS^{-} - valence change between end of acquisition and habituation phase for the conditioned stimulus associated with no aversive stimulus. See Equation 1 for more details. CS^{+}_{pain} – contingency ratings of the conditioned stimulus associated with pain, CS^{+}_{tone} – contingency ratings of the conditioned stimulus associated with aversive tone. A similar table (Table 1) can be found in the main text with only the included participants in the model development and validation (after exclusion).

Discovery sample					
CS	timepoint	N	mean	sd	se
minus	Hab	25	36.56	21.16	4.23
pain	Hab	25	37.8	17.77	3.55
tone	Hab	25	40.84	18.8	3.76
minus	Acq1	25	37.68	22.15	4.43
pain	Acq1	25	45.04	23.27	4.65
tone	Acq1	25	50.16	19.86	3.97
minus	Acq2	25	33	22.79	4.56
pain	Acq2	25	53.12	20.24	4.05
tone	Acq2	25	51	22.65	4.53
minus	Acq3	25	33.04	22.46	4.49
pain	Acq3	25	58.72	16.91	3.38
tone	Acq3	25	53.08	21.84	4.37
minus	Acq4	25	35.48	24.89	4.98
pain	Acq4	25	61.32	17.15	3.43
tone	Acq4	25	55.24	21.83	4.37
Validation sample 1					
minus	Hab	26	37.04	24.93	4.89
pain	Hab	26	34.27	19.41	3.81
tone	Hab	26	38.42	22.09	4.33
minus	Acq1	26	37.12	18.70	3.67
pain	Acq1	26	42.08	24.71	4.85
tone	Acq1	26	41.77	20.16	3.95
minus	Acq2	26	33.58	21.46	4.21
pain	Acq2	26	48.65	22.68	4.45
tone	Acq2	26	45.31	19.76	3.88
minus	Acq3	26	32.69	21.97	4.31
pain	Acq3	26	49.77	20.15	3.95
tone	Acq3	26	47.15	19.90	3.90
minus	Acq4	26	32.00	23.37	4.58
pain	Acq4	26	50.35	21.83	4.28
tone	Acq4	26	48.00	20.67	4.05
Validation sample 2					
minus	Hab	23	22.74	32.62	6.80
pain	Hab	23	16.48	30.67	6.40
tone	Hab	23	11.43	21.36	4.45
minus	Acq1	23	10.39	18.87	3.93
pain	Acq1	23	55.13	40.01	8.34
tone	Acq1	23	35.61	34.46	7.18
minus	Acq2	23	11.65	20.49	4.27
pain	Acq2	23	64.04	36.99	7.71
tone	Acq2	23	53.61	33.05	6.89

Supplementary Table 5. Raw mean valence ratings for each condition and each phase. The values in the table are visualized in *Figure 1b*. Note that the validation sample 2 measured the valence on a scale with different anchors (see *Methods* for details). Acq – acquisition, Hab – habituation.

			R^2 with pain learning (%)	R^2 with model prediction (%)	Significance of partial generalization test (p)
Divergent validity	Tone related learning change	Validation sample 1	41	7.4	0.32
		Validation sample 2	63	2.6	0.82
		Merged validation sample	53	7.8	0.35
Convergent validity	Pain extinction	Validation sample 1	42	3.8	0.54
		Validation sample 2	71.6	12.8	0.62
		Merged validation sample	58	11.2	0.33
	Contingency	Validation sample 1 (CS^-)	11.6	18.5	**0.04***
		Validation sample 1 (CS^+_{pain})	26.9	0	0.82
		Validation sample 2 (CS^+_{pain})	15.8	2.9	0.54

Supplementary Table 6. Results of partial generalization test in the validation samples. Significant result suggests the investigated variable is conditionally dependent on the model prediction (see *Results* for more detail).

BASC idx	Region name	side	x	y	z	BASC idx	Region name	side	x	y	z
172	ParOcc assoc	right	43.86	-75.48	23.78	124	Amy	right	24.1	-8.05	-20.06
173	ParOcc assoc	left	-41.6	-77.99	29.65	125	Amy	left	-23.5	-6.73	-19.7
36	pIns	right	41.34	-5.45	-4.72	13	SensMot	left	-40.99	-22.62	61.9
37	pIns	left	-43.35	-9	-2.32						
89	Angular	left	-29.83	-72.86	31.94	159	r aPFC	right	42.57	42.79	10.85
85	Cer V	left	-15.13	-45.74	-23.64	33	pCing	left	-19.46	-64.31	8.29
86	Cer V	right	14.5	-45.67	-23.53	34	pCing	right	19.72	-62.17	8.27
31	Cer VII	left	-28.67	-77.97	-50.46	137	Coll s	right	28.11	-39.22	-14.03
32	Cer VII	right	20.34	-80.48	-46.53	138	Coll s	left	-27.97	-44.01	-11.67
196	iParietal	left	-50.28	-53.65	39.49	203	Marg s	right	9.89	-28.03	45.72
						204	Marg s	left	-10.14	-32.46	48.84
147	aIns	right	37.85	22.87	4.84	75	Vis assoc	right	33.76	-81.65	18.61
148	aIns	left	-34.84	22.92	5.16	76	Vis assoc	left	-33.24	-87.73	19.12
151	aPFC	right	15.94	61.96	13.69	153	STG	left	-57.82	-4.19	-1.73
152	aPFC	left	-15.82	63.7	12.61	154	STG	right	57.9	-6.27	-0.14
43	Put	left	-27.82	-0.73	3.82	27	Vis assoc	left	-16.04	-96.35	14.02
44	Put	right	27.61	-3.86	4.46	28	Vis assoc	right	14.62	-94.47	15.27
126	dPCC	left	-13.93	-48.24	7.2	132	sParietal	right	21.08	-52.05	64.17
127	dPCC	right	11.13	-49.89	7	133	sParietal	left	-25.29	-51.44	63.42

Supplementary Table 7. Regions of predictive connections.

The table lists the left and right portions separately of all predictive connections in the RCPL signature. The table is sorted in a similar way as in *Table 3* in the main text. Region name – custom defined name of the regions; BASC idx – the index of the region from the BASC atlas ROI parcellation (MIST_ROI); x,y,z: "Center of Mass. We report the center of mass of the parcel in MNI coordinates.[8]";side – indicate the sidedness of the region; ParOcc- parieto occipital association area; Amy- amygdala; pIns- posterior insula; SensMot – sensorimotor area; Angular – angular region; r aPFC – right anterior prefrontal cortex; Cer V – cerebellar lobules; pCing – posterior cingulum; Cer VII – cerebellar lobule; Coll s – collateral sulcus; iParietal – inferior parietal lobule; Marg s – marginal sulcus; aIns – anterior insula; Vis assoc – visual associative area; aPFC – anterior prefrontal cortex; STG – superior temporal gyrus; Put – putamen; dPCC – dorsal posterior cingulate cortex; sParietal – superior parietal lobule.

1. Schlitt, F. *et al.* Impaired pain-related threat and safety learning in patients with chronic back pain. (2021) doi:10.1097/j.pain.0000000000002544.
2. Forkmann, K., Wiech, K., Schmidt, K., Schmid-Köhler, J. & Bingel, U. Neural underpinnings of preferential pain learning and the modulatory role of fear. *Cerebral Cortex* (2023) doi:10.1093/cercor/bhad236.
3. Pawlik, R. J. *et al.* Inflammation shapes neural processing of interoceptive fear predictors during extinction learning in healthy humans. *Brain Behav Immun* **108**, (2023).
4. Poldrack, R. A., Huckins, G. & Varoquaux, G. Establishment of Best Practices for Evidence for Prediction: A Review. *JAMA Psychiatry* vol. 77 Preprint at https://doi.org/10.1001/jamapsychiatry.2019.3671 (2020).
5. Gallitto, G. *et al.* External validation of machine learning models with adaptive sample splitting. *BioRxiv* (2023).
6. Koenen, L. R. *et al.* Associative learning and extinction of conditioned threat predictors across sensory modalities. *Commun Biol* **4**, (2021).
7. Spisak, T. Statistical quantification of confounding bias in machine learning models. *Gigascience* **11**, (2022).
8. Urchs, S. *et al.* MIST: A multi-resolution parcellation of functional brain networks. *MNI Open Res* **1**, (2019).
