# Supplementary material for: An externally validated resting-state brain connectivity signature of pain-related learning
Source: Commun Biol. 2024 Jul 17;7:875. doi: 10.1038/s42003-024-06574-y (PMC11255216; doi:10.1038/s42003-024-06574-y)
Supplement: Supplementary file 3 — Reporting Summary [file 42003_2024_6574_MOESM3_ESM.pdf]

Corresponding author(s): Balint Kincses

Last updated by author(s): Jun 24, 2024

Reporting Summary

Nature Portfolio wishes to improve the reproducibility of the work that we publish. This form provides structure for consistency and transparency in reporting. For further information on Nature Portfolio policies, see our Editorial Policies and the Editorial Policy Checklist.

Statistics

For all statistical analyses, confirm that the following items are present in the figure legend, table legend, main text, or Methods section.

n/a	Confirmed	
☐	☒	The exact sample size (n) for each experimental group/condition, given as a discrete number and unit of measurement
☐	☒	A statement on whether measurements were taken from distinct samples or whether the same sample was measured repeatedly
☐	☒	The statistical test(s) used AND whether they are one- or two-sided<br>*Only common tests should be described solely by name; describe more complex techniques in the Methods section.*
☐	☒	A description of all covariates tested
☐	☒	A description of any assumptions or corrections, such as tests of normality and adjustment for multiple comparisons
☐	☒	A full description of the statistical parameters including central tendency (e.g. means) or other basic estimates (e.g. regression coefficient) AND variation (e.g. standard deviation) or associated estimates of uncertainty (e.g. confidence intervals)
☐	☒	For null hypothesis testing, the test statistic (e.g. F, t, r) with confidence intervals, effect sizes, degrees of freedom and P value noted<br>*Give P values as exact values whenever suitable.*
☒	☐	For Bayesian analysis, information on the choice of priors and Markov chain Monte Carlo settings
☒	☐	For hierarchical and complex designs, identification of the appropriate level for tests and full reporting of outcomes
☐	☒	Estimates of effect sizes (e.g. Cohen's d, Pearson's r), indicating how they were calculated

Our web collection on statistics for biologists contains articles on many of the points above.

Software and code

Policy information about availability of computer code

Data collection	Discovery and validation study 1: Presentation software (http://www.neurobs.com) for behavior paradigm; software of heat stimulator device (Pathway, MEDOC); software Audacity 1.3.10-beta (http://www.audacity.sourceforge.net/<br>Validation study 2: Presentation software (http://www.neurobs.com); software barostat system (protocol plus 6.7R, 2004, G&J Electronics); software Audacity 1.3.10-beta (http://www.audacity.sourceforge.net/
Data analysis	Questionnaire data digitalizing with Microsoft Excel and processed within python (https://github.com/kincsesbalint/paintone_rsn).<br>Prerocessing MRI data with the containerized version of the in-house built "RPN-signature" pipeline (https://github.com/spisakt/RPN-signature, version 0.2.6).<br>The processing pipeline utilizes FSL (v6.0.1), AFNI (16.2.07), nipype (v1.1.9), ANTs (v2.2.0), python packages: scipy=1.1.0,scikit-learn=0.19.1, nilearn==0.5.0, seaborn==0.9.0, nipype==1.1.9, matplotlib=2.2.2, pandas=0.23.4, libxml2=2.9.8, libxslt=1.1.32, graphviz=2.40.1, traits=4.6.0, statsmodels=0.9.0.<br>Confounder analysis uses the mlconfound software package (https://mlconfound.readthedocs.io/en/latest/).<br>The full analysis pipeline with additional detailed information can be found here: https://github.com/kincsesbalint/paintone_rsn.

For manuscripts utilizing custom algorithms or software that are central to the research but not yet described in published literature, software must be made available to editors and reviewers. We strongly encourage code deposition in a community repository (e.g. GitHub). See the Nature Portfolio guidelines for submitting code & software for further information.

Data

Policy information about availability of data

All manuscripts must include a data availability statement. This statement should provide the following information, where applicable:

- Accession codes, unique identifiers, or web links for publicly available datasets
- A description of any restrictions on data availability
- For clinical datasets or third party data, please ensure that the statement adheres to our policy

All codes and processed data can be found in the project’s github page (https://github.com/kincsesbalint/paintone_rsn).
The model is available on the project's page (https://osf.io/b8znd/). The preprocessing steps and the model application can be used within the openly available containerized version of the used software tools (https://github.com/kincsesbalint/paintone_rsn) on BIDS dataset. The raw MRI data is avaialable in the project's OSF site in BIDS format (https://osf.io/b8znd/files/osfstorage).

Human research participants

Policy information about studies involving human research participants and Sex and Gender in Research.

Reporting on sex and gender	Sex was determined based on self-report.<br>Discovery sample: age(mean(SD))= 24.2 (3.9); sex(%female)=44%<br>Validation sample 1: age(mean(SD))=25.7 (4.3); sex(%female)=62%<br>Validation sample 2: age(mean(SD))=25.4 (3.7); sex(%female)=57%<br>Disaggregated sex and gender data was not collected.
Population characteristics	Healthy young volunteers were recruited by local advertisement in all samples.<br>Discovery sample: age(mean(SD))= 24.2 (3.9); sex(%female)=44%<br>Validation sample 1: age(mean(SD))=25.7 (4.3); sex(%female)=62%<br>Validation sample 2: age(mean(SD))=25.4 (3.7); sex(%female)=57%
Recruitment	Participants were recruited via advertisements and received monetary compensation.
Ethics oversight	The study was conducted in accordance with the Declaration of Helsinki and approved by the local ethics committees. All participants gave written informed consent before testing.

Note that full information on the approval of the study protocol must also be provided in the manuscript.

Field-specific reporting

Please select the one below that is the best fit for your research. If you are not sure, read the appropriate sections before making your selection.

☒ Life sciences ☐ Behavioural & social sciences ☐ Ecological, evolutionary & environmental sciences

For a reference copy of the document with all sections, see nature.com/documents/nr-reporting-summary-flat.pdf

Life sciences study design

All studies must disclose on these points even when the disclosure is negative.

Sample size	Discovery study: Sample size was calculated as described in K. Forkmann, K. Wiech, K. Schmidt, J. Schmid-Köhler, U. Bingel, Neural underpinnings of preferential pain learning and the modulatory role of fear. Cerebral Cortex (2023), doi:10.1093/cercor/bhad236<br>Sample sizes for the validation sample 1 were determined a-priory based on the expected prediction accuracy provided by nested cross-validation and were pre-registered before acquisition (https://osf.io/b8znd/).<br>Validation study 2 has been already run (retrospective data collection after model preregistration), all subjects with behavior and MRI data were shared.
Data exclusions	25 participants were excluded from the total of 99 participants due to preregistered criteria such as missing behavior or MRI data, incidental findings in MRI, falling asleep during the resting state measurement (self report), and in scanner motion limits.<br>More details on the exclusion and inclusion criteria of participants were described in the original studies (K. Forkmann, K. Wiech, K. Schmidt, J. Schmid-Köhler, U. Bingel, Neural underpinnings of preferential pain learning and the modulatory role of fear. Cerebral Cortex (2023), doi:10.1093/cercor/bhad236.; R. J. Pawlik, L. Petrakova, A. Cueillette, K. Krawczyk, N. Theysohn, S. Elsenbruch, H. Engler, Inflammation shapes neural processing of interoceptive fear predictors during extinction learning in healthy humans. Brain Behav Immun. 108 (2023), doi:10.1016/j.bbi.2022.12.010)
Replication	The data and software used here can be found in public repositories (see Data above).<br>The findings of the model prediction are validated on independent validation studies which suggest a robust and reproducible finding, given the heterogeneity of the different studies.

Randomization	A continous covariate was used as a target of our predictive model (pain-related differential valence change), therefore no experimental groups and no randomization were used. External validation was utilized and interpreted to improve generalizability and attenuate overfitting.
Blinding	Blinding was not relevant in our study. The resting state MRI measurement always preceded the behavior task. The researcher and the participant were not aware about the actual pain-related differential valence change. In discovery sample and validation study 1, participants were informed about the possible association between the CS and US.

Reporting for specific materials, systems and methods

We require information from authors about some types of materials, experimental systems and methods used in many studies. Here, indicate whether each material, system or method listed is relevant to your study. If you are not sure if a list item applies to your research, read the appropriate section before selecting a response.

Materials & experimental systems

n/a	Involved in the study
☒	☐ Antibodies
☒	☐ Eukaryotic cell lines
☒	☐ Palaeontology and archaeology
☒	☐ Animals and other organisms
☒	☐ Clinical data
☒	☐ Dual use research of concern

Methods

n/a	Involved in the study
☒	☐ ChIP-seq
☒	☐ Flow cytometry
☐	☒ MRI-based neuroimaging

Magnetic resonance imaging

Experimental design

Design type	Resting state fMRI
Design specifications	Resting state fMRI followed by the behavior task on the same day in Discovery sample 1 and validation study 2 (in-scanner), and with a median 3 days in validation study 1 (out-of-scanner). The length of the rs-FMRI was 10 minutes for Discover sample and validaiton sample 1 and 8 minutes for validation study 2 (see supplementary table 1).
Behavioral performance measures	No behavioral data was measured during the resting state MRI scan. After the resting state measurement, participants performed a differential conditioning paradigm. We characterize pain-related learning in terms of valence change.

Acquisition

Imaging type(s)	functional
Field strength	3T
Sequence & imaging parameters	Discovery study and validation sample 1: GE 2D EPI sequence, 38 slices, resolution 2.5 x 2.5 x 3 mm, TE 28.0 ms, TR 2300 ms, GRAPPA 2, distance factor 15%, 260 volumes<br>Validation sample 2: GE 2D EPI sequence, 46 slices, 3 x 3 x 3 mm, TE:30 ms, TR: 2500 ms, GRAPPA: 3, 192 volumes
Area of acquisition	whole brain scan

Diffusion MRI ☐ Used ☒ Not used

Preprocessing

Preprocessing software	Prerocessing MRI data: the containerized version of the in-house built "RPN-signature" pipeline (https://github.com/spisakt/RPN-signature, version 0.2.6).<br>The processing pipeline utilizes FSL (v6.0.1), AFNI (16.2.07), nipype (v1.1.9), ANTs (v2.2.0), python packages: scipy=1.1.0,scikit-learn=0.19.1, nilearn==0.5.0, seaborn==0.9.0, nipype==1.1.9, matplotlib=2.2.2, pandas=0.23.4, libxml2=2.9.8, libxslt=1.1.32, graphviz=2.40.1, traits=4.6.0, statsmodels=0.9.0.
Normalization	Anatomical data was normalized with ANTs, see the source code of the "RPN-signature" pipeline (https://github.com/spisakt/RPN-signature, version 0.2.6) for parameters.<br>Functional data was co-registered to the anatomical image with FSL Flirt Boundary-based registration (BBR).<br>Analysis was done in native functional space by brain-atlas individualization.
Normalization template	The 1mm-resolution MNI152 Template was used.
Noise and artifact removal	Motion correction (FSL mcflirt) , nuisance regression (Friston-24-expansion), temporal SNR-based compcor correction with 5

Noise and artifact removal	components, bandpass filtering (AFNI bandpass, 0.08-0.008Hz), despiking, and scrubbing of high motion time frames (framewise displacement FD>0.15)
Volume censoring	We preregistered our thresholds after model discovery. Potentially motion-contaminated time-frames were scrubbed (e.g.: dropped from the data), defined by a conservative preregistered FD>0.15mm threshold. If the mean FD exceeded 0.15mm, or when more then 25% of frames were scrubbed, participants were excluded from further analysis.

Statistical modeling & inference

Model type and settings	The following steps were implemented in the model discovery phase with a nested cross-validation approach in scikit-learn: pre-selecting K best feature (strongest association with the target variable) and a Ridge regression model. Two free parameters of the model were the number of pre-selected features (k) and the regularization strength (alpha). An optimized grid-search procedure with the negative mean squared error as cost function. The range of K values were between 10 and 200 with an increment of 5. Alpha values could take [.001, .005, .01, .05, .1, .5, 1, 10].<br>Nested cross-validation was applied to overcome any information leakage. Separate samples were used for model validation.<br><br>Important to note that fMRI preprocessing was independent between subjects, thus it was not included in the cross-validation approach.
Effect(s) tested	Prediction accuracy in the validation sample was tested with permutation tests.

Specify type of analysis: ☐ Whole brain ☒ ROI-based ☐ Both

Anatomical location(s)	A functional brain parcellation (BASC atlas: derived with the BASC (data-driven) algorithm) with 122 non-overlapping functional regions and additionally the global grey matter signal
Statistic type for inference (See Eklund et al. 2016)	Does not apply.
Correction	No mass-univariate analysis.

Models & analysis

n/a	Involved in the study
☐	☒ Functional and/or effective connectivity
☒	☐ Graph analysis
☐	☒ Multivariate modeling or predictive analysis

Functional and/or effective connectivity	partial correlation
Multivariate modeling and predictive analysis	Whole-brain functional atlas-based resting-state connectivity was used as the feature space (n=7503 features per participant) and individual pain-related differential valence change as our target (preregistered before the acquisition of the external validation samples). A feature selection step (select k best - strongest relationship with the target variable) and a regularized regression (Ridge) fitting was used in the model fitting.
